# Collectanea Medica: Consisting of Anecdotes, Facts, Extracts, Illustrations, &c.

**Published:** 1823-02

**Authors:** 


### COLLECTANEA MEDICA:

CONSISTING OF

ANECDOTES, FACTS, EXTRACTS, ILLUSTRATIONS, &c.

*Relating to the History or the Art of Medicine, and the Collateral Sciences.*

Floriferis, ut apes, in saltibus omnia libant,

Omnia nos, itidem, depascimur aurea dicta.

WE extract the following *new* articles from the fifth edition of Dr. PARIS's Pharmacologia, recently published; conceiving that they may prove interesting to such of our readers as have already procured the preceding editions.

### ART. I.—*Acidum Hydro-Cyanicum. Hydro-cyanic Acid. Prussic Acid.*

THIS peculiar acid exists in a great variety of native combinations in the vegetable kingdom, and imparts to them certain properties which have been long known, and esteemed in medicine. It is, however, only lately that it has been administered in its simple, but diluted, form. As few practitioners will choose to prepare the acid, it seems unnecessary in the present work to dwell upon the merits of the different processes which have been proposed for its preparation: for a full account of them, as well as for other details of importance, the practitioner is advised to consult a work by Dr. Granville, entitled "An Historical and Practical Treatise on the use of Prussic Acid. Second edition. London, 1820."

*Qualities.*—A colourless transparent liquid, although it occasionally exhibits a yellow tinge; odour like that of bitter almonds; taste bitterish and peculiar: these properties, however, are soon lost by exposure to air and light, and the acid undergoes spontaneous decomposition.

*Chem. Composition.*—The true nature of prussic acid was not ascertained until 1815, when Gay-Lussac presented to the Royal Institute of France a memoir which at once developed its real chemical constitution; and it is now admitted to consist of a peculiar gaseous and highly inflammable compound of carbon and nitrogen, to which the name *cyanogene* has been assigned, and hydrogen; the latter body acting as the acidifying principle, whence the term *hydro-cyanic* acid is well contrived to express its composition. The medicinal acid, however, contains but a small proportion of this concentrated compound: according to M. Majendie, one part of the acid of Gay-Lussac and eight parts and a half of water, by weight, constitute the preparation which should be used in medicine. Dr. Ure, who has lately taken considerable pains upon this subject, has constructed a table exhibiting the relations between the specific gravities and quantities of real acid, in preparations of different strength: from these experiments it would appear that an acid of specific gravity 0.996 or 0.997 is such as is usually prescribed in medicine.

*Medical Uses.*—In a sufficient dose, hydrocyanic acid instantly destroys life by extinguishing the nervous energy of the body; but it has at the same time been observed, that animals submitted to its action would often continue to breathe for several hours freely, and to circulate their blood, although no trace of sensibility or muscular contractility could be found after its application. This remarkable property of extinguishing the general sensibility, without any ostensible injury to respiration and circulation, naturally led to a belief that the hydrocyanic acid, or prussic acid, might be advantageously used in cases of excessive sensibility and irritation, particularly when these two morbid states are likely to affect either the respiratory organs or the circulation generally. This kind of analogical reasoning, it is said, induced Professor Brera, ten years ago, to administer it in cases of high pulmonary and other inflammations, in doses of four drops twice a-day; when, as we are told, the violence of the disease was quickly subdued. The remedy, however, does not appear to have excited much attention, until after the first essay of Dr. Majendie, who deserves whatever credit may belong to its introduction. Seven years of trial have elapsed, and the general sense of the medical profession with respect to its utility may now be collected. As a palliative in certain spasmodic coughs, there is reason for supposing that it *may sometimes* be useful; but, in that species of pulmonary irritation for which it was at first so greatly extolled, I will venture to assert that it is far inferior in efficacy to well-directed doses of Conium. But there is another class of diseases in which its exhibition is said to prove useful, in dyspeptic affections attended with heart-burn; where it is supposed to be capable of reducing the morbid irritability of the stomach, and thereby of enabling the juices of that organ to be more slowly secreted and of a more healthy character. Dr. Elliotson has published the result of his treatment of stomach complaints with this medicinal agent, and would appear to appropriate to himself the merit of originating the practice; a claim which Mr. Thomson, in the third edition of his Dispensatory, refuses to concede. As a local remedy, prussic acid has also received no small share of commendation, and it has been said that it is the only application that can be depended upon for allaying the cutaneous irritation so frequently attendant upon certain impetiginous affections.

*Forms of Exhibition.*—It may be conveniently administered in any liquid vehicle, as distilled water, camphor mixture, or in some vegetable infusion. A question has lately arisen, whether the effects of the prussic acid might not be more conveniently ensured by the administration of some vegetable in which it exists as a native ingredient: a company of associated physicians, surgeons, and naturalists, at Florence, have accordingly expressed their joint opinion, that the essential oil of the Prunus lauro cerasus is to be preferred, in medical practice, to all other preparations which contain the hydro-cyanic acid; for, say they, unlike the distilled water of the plant, and pure prussic acid, it contains the same proportion of active matter and of the same power, whether recently prepared or not; whether made in one place or another; or whether it has been exposed or not to air, light, or heat. They are also of opinion that olive-oil forms the best vehicle for its exhibition, in the proportion of one ounce to twelve drops of the essential oil. Other practitioners again prefer Laurel water, made by distilling two drachms of the fresh leaves chopped, with four ounces of water, recommitting the distilled water twice afterwards on the same quantity of fresh leaves, and making ultimately four ounces of the menstruum; of which from ♏ xxx. to f ʒ j. every six hours may be given, until a sedative effect is produced.

*Incompatible Substances.*—Hydro-cyanic acid is decomposed by most of the oxydes usually employed in medicine, particularly by those of mercury and antimony. The alkalies do not appear to diminish its efficacy. Nitrate of silver, and the salts of iron, occasion precipitates; nor ought the sulphurets, the mineral acids, or chlorine, to enter with it into prescriptions.

*Dose.*—Of the medicinal hydro-cyanic acid, ♏ ij.—viij. There is, however, considerable difficulty with regard to the strength of the dilute acid employed in medicine, since the density is a criterion of greater nicety than can be conveniently used by the majority of practitioners: in fact, as Dr. Ure has observed, the liquid at 0.996 contains about double the quantity of real acid, which it does at 0.998. Dr. Ure has accordingly proposed another test of the strength of this powerful and dangerous medicine, which is not only easier in use, but more delicate in its indications; it is as follows:—To 100 grains, or any other convenient quantity of the prussic acid, contained in a small phial, add in succession small quantities of the peroxide of mercury, (the common red precipitate of the shops,) in fine powder, till it ceases to be dissolved on agitation. The weight of the red precipitate taken up, being divided by four, gives a quotient representing the quantity of real prussic acid present. By weighing out before-hand, on a piece of paper, or a watch-glass, forty or fifty grains of the peroxide, the residual weight of it shows at once the quantity expended. The operation may always be completed in five minutes, for the red precipitate dissolves as rapidly in the dilute prussic acid, with the aid of slight agitation, as sugar dissolves in water.

*Adulterations.*—If, says Dr. Ure, the presence of muriatic acid be suspected, then the specific gravity of the liquid, compared with the gravity of the peroxide dissolved, will show how far the suspicion is well founded: thus, if 100 grains of acid, specific gravity 0.996, dissolve more than 12 grains of the red precipitate, we may be sure that the liquid has been contaminated with muriatic acid. Nitrate of silver, in common cases so valuable a re-agent for muriatic acid, is unfortunately of little use here; for it gives with prussic acid a floculent white precipitate, soluble in water of ammonia, and insoluble in nitric acid, which may easily be mistaken, by common observers, for the chloride of that metal. But the difference in the volatility of prussiate and muriate of ammonia may be had recourse to with advantage; the former exhaling at a very gentle heat, the latter requiring a subliming temperature of about 300° Fah. After adding ammonia in slight excess to the prussic acid, if we evaporate to dryness at a heat of 212°, we may infer, from the residuary sal ammoniac, the quantity of muriatic acid present.

*Antidotes.*—To counteract the poisonous effect of prussic acid, Orfila recommends, after full vomiting has been excited, the exhibition of three or four spoonsful of oil of turpentine, in the infusion of coffee, at intervals of half an hour. M. Virey conceives that sulphate of iron in solution is the best antidote, he having observed that the salt restored a cow that was nearly killed by the essential oil of bitter almonds. When an overdose has been taken, hot brandy and water, and the ammoniated tincture of iron, are recommended by Mr. Thomson. To the former I would rely with much greater confidence than upon the latter antidote; or, in other words, it is from vital agents counteracting its sedative influence, rather than from chemical substances changing its composition, that we can expect any benefit upon such an occasion.

It has very lately been asserted by Dr. MACLEOD, that the hydrocyanic acid occasionally produces soreness of the gums and a disposition to ptyalism: this, if true, is a very remarkable fact, and well deserves attentive consideration.

---

In the Number of this Journal for October 1821, I related the history of two individuals, both of whom became affected with soreness of the mouth and ptyalism during the use of this medicine. In both it was discontinued, and its use resumed several weeks after these symptoms had disappeared; in both the affection of the gums returned. Yet I have never met with any other instance of this kind, although I have been on the watch for it, and have since employed the hydrocyanic acid in at least two hundred cases, principally diseases of the stomach. Dr. GRANVILLE, in his Historical Retrospect for January 1822, mentions his having witnessed a similar effect from this medicine.—R. M'L.

---

ART. II.—*Tiglii Oleum, (Oil of Tiglium.) Croton Tiglium, (Oleum e Seminibus expressum.)*

THE croton tiglium is a native of the island of Ceylon, and is found in Malabar, China, Cochinchina, and the Molucca islands. Every part of the plant would seem to be endowed with medicinal activity: the root acts as a drastic purgative, and when pulverized, and exhibited in the dose of a few grains, is considered, at Amboyna and Batavia, as a specific for dropsy; the wood (*lignum Pavanæ*) produces, when administered in small doses, a diaphoretic effect, and in larger ones it proves drastic; the leaves are also purgative, and, when dried and powdered, are supposed to afford an antidote against the bite of the Cobra de Capella. The seeds, however, are the parts which have been more generally employed in medicine, the effects of which appear to have been well known for nearly a thousand years. They were early introduced into Europe, and long known under the names of Grana Molucca, Tiglii Grana, and Grana Tiglia. It appears that they were at first very frequently administered, but their extreme acrimony and violence, and probably the accidents which arose from their injudicious use, soon banished the article from medical practice. In India, however, these seeds are still employed as an effectual purgative, after first undergoing the process of roasting or baking, for the purpose of removing the shell, rendering the nut pulverulent, and at the same time of moderating its acrimonious qualities. The expressed oil of these seeds does not appear to have been obtained in a separate form until a later period. Lemery speaks of it; and Geoffroy, in directing its dose, cautions us against giving more than ʒj! —he probably meant a drop. Its use has very lately been revived, and there can be little doubt but that, under proper restrictions, it may become a valuable acquisition to the practitioner. The profession is indebted for its late introduction, or rather revival, to Mr. E. Conwell, of the East-India Company's medical service, on the Madras establishment, who, having for many years prescribed it with advantage, introduced a quantity of it for trial in London, through the medium of his friend, Mr. Short, of Ratcliff Highway.

*Qualities.*—This expressed oil has a yellow colour, a faint odour, and an acrid taste: these qualities, however, will be found to vary in different samples; but the fact, as Dr. Nimmo has justly observed, may be fairly explained, without suspecting the existence of any fraud, by supposing that the seeds have undergone a different degree of torrefaction, in order to separate the oil from the farinaceous part.

*Chemical Composition.*—The recent experiments of Dr. Nimmo have very satisfactorily shown that this oil consists of 45 parts of an acrid purgative principle, and 55 of a fixed oil resembling that of olives, and not possessed of any cathartic property. The acrid principle appears to reside in a resinous matter, soluble in alcohol and sulphuric æther, and in volatile and fixed oils. I have lately repeated some of Dr. Nimmo's experiments on a recently imported sample of oil, and with similar results. The acrid principle appears to bear a strong analogy to that which I separated from elaterium; and, as I gave to this latter principle the term Elatin, it seems to me that we might, with much propriety, apply the name Tiglin to the former, especially as it does not appear to possess any of the characters and habitudes of a salifiable basis; at all events, the adoption of such a term will obviate the necessity of circumlocution in our descriptions.

*Solubility.*—By alcohol the oil undergoes a ready decomposition the tiglin is dissolved, together with a very minute quantity of the oily part. Ether and oil of turpentine dissolve the whole; a fact which enables us, by digesting the seeds in these menstrua, to obtain the article in as genuine, and certainly in a much more uniform, condition, than by the processes of torrefaction and expression, as practised in India. For this fact we are also indebted to Dr. Nimmo.

*Medical Uses.*—As far as I have been able to ascertain the fact, this oil does not appear to produce any effects which cannot be commanded by other drastic purgatives: its value depends upon the facility with which it may be administered; in some cases it is amply sufficient to touch the tongue; in others, a drop or two will be required. In maniacs, and in cases where the administration of bulky medicines is extremely difficult, it would seem to offer a decided advantage.

*Forms of Exhibition.*—It has been usually given in this country in the proportion of from one to two drops, in the form of pills. Dr. Nimmo's discovery with respect to the chemical composition of the oil, very naturally suggested to him the mode of administering it in the form of an alcoholic tincture, (Tinctura tiglii,) and he has found by experience that such a preparation furnishes the means of readily apportioning the dose to the various circumstances of the case. Thus he found that, in administering a tincture in doses equivalent to the number of drops decomposed, the same effects were produced as have been attributed to the entire oil.

*Adulterations.*—Much has been said upon the fraudulent admixture of this comparatively expensive article with the cheaper fixed oils, and we believe with much truth; a circumstance which will, of necessity, prevent the general use of the article, and occasion very different reports with respect to its value and activity. Dr. Nimmo, however, proposes a method of detecting such adulteration, by a process suggested by the results of his experiments upon its composition, and whose rationalé will be easily understood after the chemical history that has been just presented.

"Let a very light phial be counterpoised in an accurate balance: pour into it 50 grains of the suspected oil, add alcohol, (which has been previously digested upon olive-oil,) agitate them well, pour off the solution and add more alcohol as before, until the dissolved portion is diffused in such a proportion of alcohol that each half-drachm measure shall contain equal to one dose of the oil of tiglium for an adult. By afterwards placing the phial near a fire, to evaporate what remains of the alcohol in the bottle, if the residuum be to that which has been abstracted by the alcohol as 55 to 45, the oil is genuine. If olive, or any other oil little soluble in alcohol, has been employed as the adulterating agent, it is evident that the residuum will be in a larger proportion; but, should castor-oil have been employed for that purpose, the proportion of the residuum will be smaller even than in the genuine medicine."

